# Maternity waiting homes in Liberia: Results of a countrywide multi-sector scale-up

**DOI:** 10.1371/journal.pone.0234785

**Published:** 2020-06-23

**Authors:** Jody R. Lori, Joseph E. Perosky, Sarah Rominski, Michelle L. Munro-Kramer, Faith Cooper, Alphonso Kofa, Aloysius Nyanplu, Katherine H. James, G. Gorma Cole, Katrina Coley, Haiyin Liu, Cheryl A. Moyer

**Affiliations:** 1 School of Nursing, University of Michigan, Ann Arbor, MI, United States of America; 2 College of Human Medicine, Michigan State University, East Lansing, MI, United States of America; 3 Department of Obstetrics and Gynecology, University of Michigan, Ann Arbor, MI, United States of America; 4 International Rescue Committee, Sophie’s Junction, Monrovia, Liberia; 5 Bong County Health Team, Suakoko District, Liberia; 6 Department of Learning Health Sciences, University of Michigan Medical School, University of Michigan, Ann Arbor, MI, United States of America; Ghana Health Services, GHANA

## Abstract

**Objective:**

Descriptions of maternity waiting homes (MWHs) as an intervention to increase facility delivery for women living in remote geographic areas dates back to the 1950s, yet there is limited information on the scale-up and sustainability of MWHs. The objective of this study was to describe the evolutionary scale-up of MWHs as a component of health system strengthening efforts and document the successes, challenges, and barriers to sustainability in Liberia.

**Methods:**

Data were collected from a national sample of 119 MWHs in Liberia established between 2010–2018. The study used a mixed method design that included focus group discussions, individual interviews, logbook reviews, and geographic information systems. Qualitative data were grouped into themes using Glaser’s constant comparative method. Quantitative data were analyzed using negative binomial regression to measure the differences in the counts of monthly stays at facilities with different funding sources and presence of advisory committee. Additionally, each MWH was geo-located for purposes of geo-visualization.

**Results:**

In the years since the original construction of five MWHs, an additional 114 MWHs were constructed in 14 of the 15 counties in Liberia. Monthly stays at facilities funded by community were 2·5 times those funded by NGOs (IRR, 2·46, 95% CI 1·33–4·54). Attributes of sustainability included strong local leadership/active community engagement and community ownership and governance.

**Conclusion:**

Success factors for scale-up and sustainability included strong government support through development of public policy, local and county leadership, early and sustained engagement with communities, and self-governance. A multi-pronged approach with strong community engagement is key to the scale-up and sustainability of MWHs as an intervention to increase facility delivery for women living the farthest from a healthcare facility.

## Introduction

Decreasing maternal mortality has become a worldwide priority. The Millennium Development Goals (MDGs) ushered in a 43% reduction in maternal deaths between 1990–2015 through efforts to improve the quality of care and strengthen health systems [1]. In many low- and middle-income countries (LMICs), vast distances between communities and healthcare facilities, challenging terrain, and a lack of transportation often restrict access to skilled birth attendants for the most vulnerable women [2]. Access to a skilled birth attendant remains one of the most important issues to improve maternal outcomes [3].

One aspect of health system strengthening efforts has been the introduction of maternity waiting homes (MWHs). Maternity Waiting Homes are facilities that house women in the last few days or weeks of pregnancy, offering easy access to a nearby healthcare facility capable of providing emergency obstetric care (EmOC) once labor begins [4]. Multiple models of MWHs are described in the literature, from risk-based models (i.e., women with risk factors are encouraged to stay in a MWH to decrease poor outcomes) to models that follow a national strategy (i.e., any pregnant woman is allowed to stay at a MWH prior to delivery) [5,6].

Providing shelter near an obstetric facility for women living in remote geographic areas prior to delivery is not a new concept [7]. Descriptions of MWHs date back to the 1950s with examples of their use on multiple continents [8–11]. In a recent Maternal Health Lancet Series, MWHs were identified as one solution to decrease maternal morbidity and mortality by bringing women living in hard-to-reach areas closer to a hospital or health center that provides EmOC [12].

### Liberian context

Liberia was designated a “fragile” country targeted by the Global Financing Facility (GFF) in 2015, and is ranked tenth in the world for maternal mortality [13]. The maternal mortality rate is estimated at 1,072 deaths per 100,000 live births, placing a woman’s lifetime risk of dying from a pregnancy-related complication at 1 in 31 [14]. According to the most recent published Liberian Demographic Health Survey, 56% of all births in the country take place in a facility, with profound rural/urban differences [15]. Distance to a health facility was identified as a barrier to facility delivery by 40% of women surveyed; thus impacting their ability to access skilled care during childbirth [15].

In 2010, a cohort study of 10 rural primary health facilities (5 with and 5 without a MWH) was conducted in Bong County, Liberia to evaluate the impact of MWHs on childbirth outcomes. Results showed a decrease in maternal and perinatal mortality in communities with a MWH compared to those without one [16]. Following the dissemination of results from this study, the Liberian Ministry of Health (MOH) identified MWHs as one critical component of advancing health system strengthening efforts to improve maternal outcomes [17].

To date, there is limited and disorganized information on the scale-up and sustainability of MWHs. The purpose of this study was to examine the scale-up of MWHs within Liberia since the original five were built to: (1) describe the evolutionary development of MWHs as a component of the larger health system strengthening efforts; (2) describe the role of MWHs to improve maternal health from a community and healthcare provider viewpoint; and (3) document the successes, challenges, and barriers to sustainability and scale-up of MWHs.

## Methods

This study used a convergent parallel mixed methods design that included qualitative data in the form of focus group discussions (FGDs), individual interviews, quantitative data retrieved from logbook reviews, and geo-location data collected through geographic information systems (GIS). Focus group discussions were conducted with community members, including chiefs, community leaders, women of reproductive age, traditional birth attendants (TBAs), women currently staying at a MWH, and male partners. Individual interviews were conducted with healthcare providers (midwives, registered nurses, and officers in charge) providing services at the rural primary healthcare facilities associated with a MWH. Logbook registries at rural health facilities with a MWH were reviewed to capture MWH usage. Additionally, each MWH was geo-located for purposes of geo-visualization [18].

Working closely with the MOH and county health teams in Liberia, we identified and traveled to all 119 MWHs currently in use, under construction, or abandoned since the original study was conducted from 2010–2013. The research was approved by ethical review boards from the University of Michigan (HUM00132812) and University of Liberia (17-09-068).

### Setting and sample

This descriptive study used a national sample from all 15 counties in Liberia. The County Health Officer in each county identified the MWHs within their jurisdiction. All but one county had at least one MWH. All MWHs were located in close proximity to a MOH supported rural health facility.

### Data collection

Data were collected between December 2017 and June 2018 by a team comprised of two US and two Liberian research assistants (RAs) fluent in the local language. Prior to field visits all team members received training through the Program for Education & Evaluation in Responsible Research and Scholarship (PEERRS), a research ethnical training program. The research team visited communities with MWHs in order of geographic proximity, calling ahead to ensure the County Health Team and local healthcare providers were aware of their arrival. All data were collected during the facility visit.

Prior to the research team’s arrival in the communities, the County Health Officer notified the Officer in Charge (OIC) at the rural primary healthcare facility associated with each MWH. The OIC coordinated with community health volunteers to inform community members/potential study participants of the research. Interested participants from the community then arrived at the health facility on the established date the team was arriving to take part in the FGD. Focus groups were comprised of a mix of men and women from the community.

Prior to FGDs and individual interviews, the RAs provided an explanation of the study and obtained verbal or written informed consent based on literacy level of the participant. All community members provided verbal informed consent, making a mark on the consent form and witnessed by the RA. All healthcare providers gave written informed consent. Participants were informed they could refuse to answer any questions or stop participation at any time. All data were de-identified and stored on an encrypted server.

A total of 115 FGDs were conducted in the local language with 8–12 community members who volunteered to participate in each FGD. Focus groups took approximately 45–60 minutes. Questions focused on the community’s role in the development and governance of the MWH, utilization and awareness of the MWH in their community, as well as barriers, challenges, and benefits. All FGDs were tape recorded, translated into English, and transcribed verbatim by a Liberian RA.

Individual interviews were conducted with 113 healthcare providers at rural primary healthcare facilities associated with a MWH. A structured interview guide was used for the individual interviews that included open-ended questions to examine components of successful models of MWHs, perceptions of how MWHs affect relationships between providers and community members, and challenges to sustainability of MWHs. Interviews lasted approximately 30–45 minutes and responses were recorded verbatim by the interviewer onto the interview guide.

Facility logbook reviews were conducted at each site, gathering information on MWH utilization. This included tallying the number of individuals who stayed at the MWH over the previous 12 months, as well as the duration of their stay.

### Data analysis

The research team of Liberian and US investigators and RAs individually reviewed all transcripts manually for general impressions. Team members met to debrief and compare interpretation. Data were coded and grouped into conceptual categories using Glaser’s constant comparative method for qualitative research [19,20]. JRL and JEP then analyzed the coded data and conceptual categories separately and together until common themes emerged. Final core themes were then reviewed and discussed by the entire team until consensus was achieved by all study authors on the final core themes [21]. To ensure trustworthiness of the data the following activities were employed throughout the study process: sampling adequacy, collecting and analyzing data concurrently, member checking, and methodological coherence [22].

Summary statistics based on mean, frequency, or range were carried out for the exploratory analysis on the main outcome variable of MWH use. We conducted negative binomial regression to measure the differences in the counts of monthly stays at facilities with different funding sources and presence of an advisory committee. Final models were adjusted for facility size (number of beds), completion year (dummy variable indicating opened during or after the funding period), and the size of community population. Incidence rate ratios (IRRs) and 95% confidence intervals (CIs) were estimated. All analyses were run in Stata, version 15·0.

## Results

Demographic data were collected on all focus group and individual interview participants. The sample included 113 healthcare providers posted to rural clinics with a MWH (midwife or nurse (n = 73), officer-in-charge (n = 37), and other (n = 3)). Demographics for FGD participants are displayed in Table 1.

**Table 1:** Demographic information of focus group participants.

Demographic Characteristic	Number of Participants	%
	*n* = 1,179	
**Age**		
Range	16–90	6.2%
Mean (SD)	41.2 (16.2)	
Unknown Age	73	
**Years in Community**		
Range	.08–90	4.4%
Mean (SD)	28.7 (20.7)	
Unknown Years in Community	52	
**Relationship Status**		
Married	858	72.8%
Divorced/Separated	124	10.5%
Widowed	23	2.0%
Single	161	13.7%
Unanswered	13	1.1%
**Number of Children**		
Range	0–19	0.6%
Mean (SD)	4.8 (3.2)	
Unanswered	7	
**Has Used MWH**		
Yes	434	36.8%
No	738	62.6%
Unanswered	7	0.6%
**Role**†		
Chief	82	7.0%
Community Leader	163	14.0%
Woman of Reproductive Age	298	25.6%
Traditional Birth Attendant	221	19.0%
Woman at MWH	196	16.8%
Male Partner	205	17.6%
**County**		
Bong	194	16.5%
Bomi	15	1.3%
Gbarplou	16	1.4%
Grand Bassa	154	13.1%
Grand Cape Mount	27	2.3%
Grand Gedeh	30	2.5%
Grand Kru	13	1.1%
Lofa	132	11.2%
Margibi	42	3.6%
Maryland	43	3.6%
Montserrado	66	5.6%
Nimba	241	20.4%
Rivercess	193	16.4%
River Gee	13	1.1%

†*n* = 1165; 14 participants did not answer.

### Scale-up of maternity waiting homes

**Fig 1:**
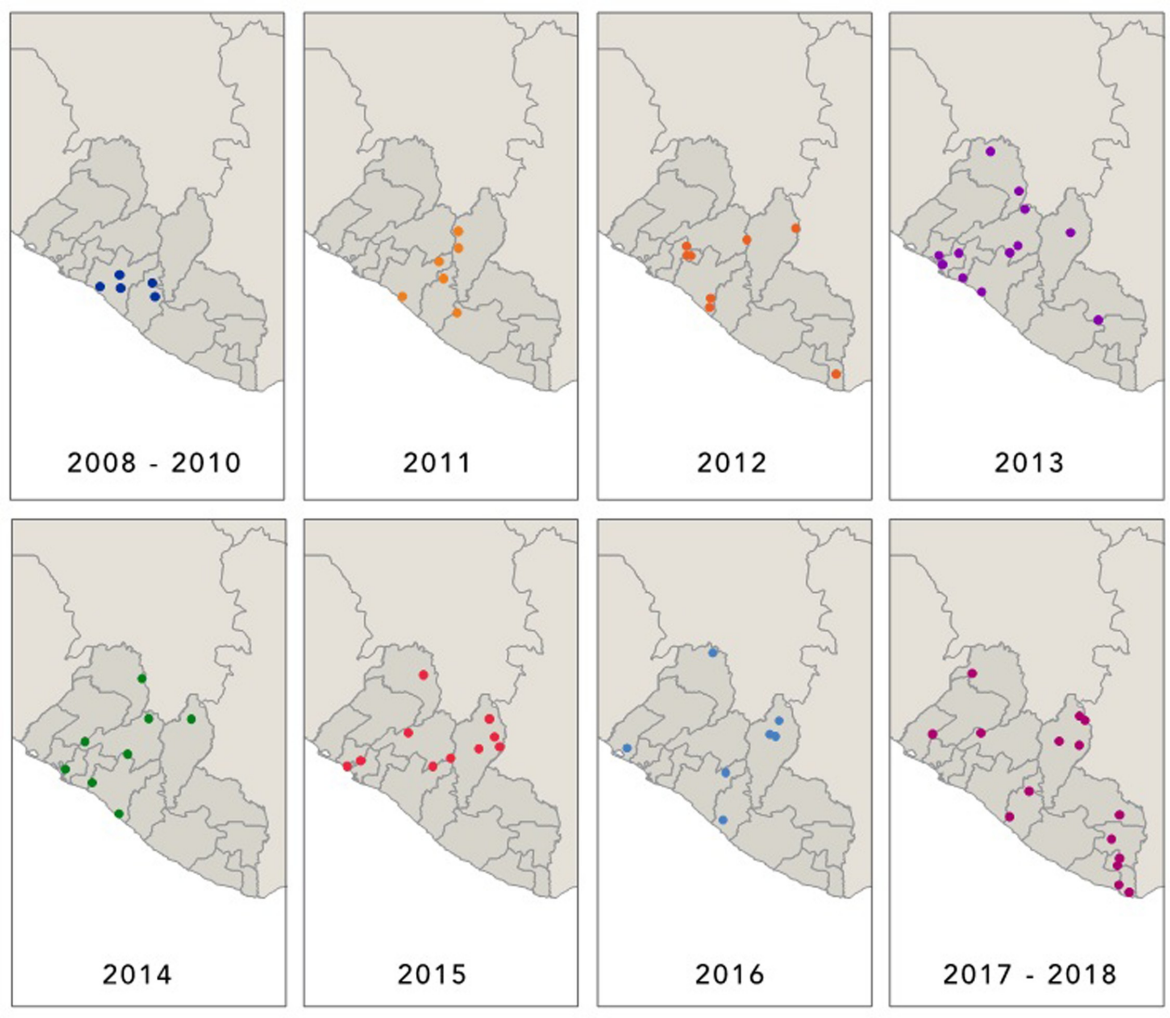
Scale up of MWHs in Liberia by year. Scale up by year of MWHs in Liberia since the first five were constructed. Construction started in the center of the country (in more populated counties) and spread first to neighboring counties and then to areas with sparser populations.

In the years since the original construction of five MWHs, an additional 114 MWHs were constructed in 14 of the 15 counties in Liberia with support from diverse funding streams and implementers. To date there are 119 MWHs in Liberia. Fig 1 depicts the scale up by year since the first five were constructed. As noted by the maps, MWH construction started in the center of the country in more populated counties and spread first to neighboring counties and then to areas with sparser populations.

**Fig 2:**
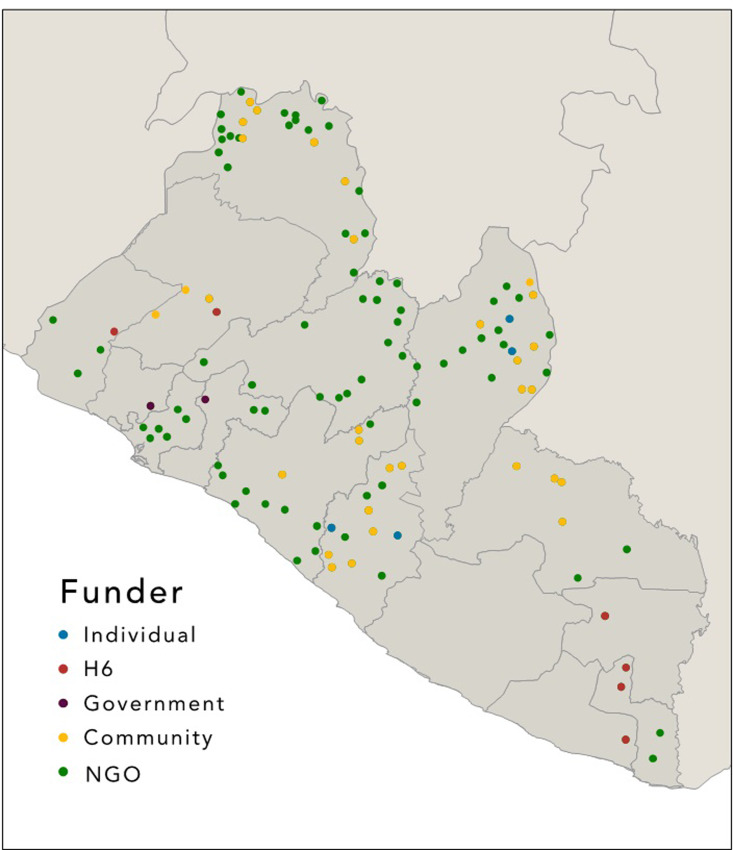
Maternity Waiting Home in Liberia by funder. Funders and implementers involved in the scale up of MWHs in Liberia (2008–2018).

Of the 119 MWHs, 54 (45·4%) were open and functional, 8 (6·7%) were currently under construction, 35 (29·4%) had started construction but ceased prior to opening, 15 (12·6%) have been repurposed mainly for staff quarters, and 7 (5·9%) MWHs were opened and later abandoned for multiple reasons. There were various funders and implementers involved in the scale up of MWHs including 72 (60·5%) by NGOs, 35 (29·4%) by the local community, 6 (5·0%) by the United Nations H6 consortium, 4 (3·4%) by local individuals, and 2 (1·7%) by the local Liberian government (see Fig 2).

The majority (n = 58, 73%) of MWHs constructed with government and NGO support were open and functioning for the expressed purpose of providing shelter for pregnant women living long distances from a health facility prior to delivery. The community and individually funded MWHs were more likely to be under construction for a longer period or to have construction stopped due to shortage of funds with only 14 (36%) open and fully functioning.

Overall, MWHs were serving between 4 and 74 catchment (μ = 18) communities with populations ranging from 988 to 35,841 in these catchment areas. The average population per MWH is 8,430 with nearly 700,000 Liberians (1,475 communities) living within a functioning MWH catchment area. The number of beds available in each MWH ranged from 1–48 with a median of 5.

Maternity waiting homes in Liberia were being used for pregnant women awaiting delivery as well as postpartum women. The average number of women using a MWH monthly ranged from 0–52 with a mean of 11. The average length of stay at MWHs was 15·9 days antenatally (range 1·0–40·0 days) and 2·8 days postpartum (range 0·5–21·0 days) addressing this most neglected period for the provision of quality care.

Monthly stays by pregnant women at MWHs funded by communities were 2·5 times those funded by NGOs (IRR, 2·46, 95% CI 1·33–4·54). For facilities with a local advisory committee providing management and oversight of the MWH, monthly stays increased 7% compared to those without one (IRR 1·07, 95% CI 0·41–2·81). See Table 2.

**Table 2:** MWH use by funder and advisory committee.

Average number of monthly stays	IRR (95% CI)
**Funder**	
NGO (reference)	1
Community	2.46 (1.33, 4.54)
Other	0.52 (0.28, 0.96)
**Advisory Committee**	
No (reference)	1
Yes	1.07 (0.41, 2.81)

### Community and healthcare provider beliefs

Three main themes emerged from the data on the role of MWHs to improve maternal health. Nearly all communities and healthcare providers held the following three beliefs about MWHs in rural Liberia: (1) MWHs reduce home deliveries; (2) MWHs reduce maternal deaths; and (3) MWHs improve relationships between health facility staff, TBAs, and communities.

#### Maternity waiting homes reduce home delivery

Several healthcare providers noted a decrease in home deliveries with construction of MWHs at their healthcare facility: “It reduced the home deliveries, complications, and maternal deaths.” Others believed MHWs reduced the number of women delivering on the way to the facility: “When the women came before they would have road and home deliveries.” Another noted: “The presence of the maternity waiting home has also discourage[d] home delivery and at the same time given pregnant women the chance to come and stay until their time for delivery.”

#### Maternity waiting homes reduce maternal deaths

Community members strongly believed that MWHs were reducing maternal deaths in their communities as noted by this participant: “The waiting home is also helping to reduce number of death[s]… as the result of women giving birth in the absence of a health facility or a waiting home.” And this father noted: “The waiting home is contributing…in the reduction of death because community health workers can go from community to community to encourage women who are pregnant to come in their ninth month and stay at the waiting home until their time for delivery.”

#### Maternity waiting homes improve relationships between health facility staff, traditional birth attendants, and communities

In communities with functioning MWHs there was a sense of better relationships between communities, TBAs, and facility staff as noted by this healthcare provider: “It brings us together to help us understand each other’s work. It helps to talk with the TBAs more often. It makes relationships with TBAs better. The TBAs are proud to bring women here. Yesterday the TBAs brought seven big bellies.” A community member noted: “It alleviates fears and makes people to know the nurses and midwives are friendly.”

### Successes and challenges to sustainability of MWHs

Successful models of MWHs in Liberia had several attributes in common. The main themes from the FGDs and individual interviews describing successful models included: 1) strong local leadership/active community engagement and 2) community ownership/governance.

#### Strong local leadership/active community engagement

There was wide diversity in engagement with communities prior to the construction of MWHs. Communities that were engaged in the initial processes of setting up and developing strategies for maintaining and using the MWH often resulted in MWHs with the highest utilization by pregnant women. One health worker noted the following:

The building of this maternity waiting home was not a secret because announcement was made all over the community… people came and had the ground breaking… the paramount chief also came and had big meeting with them…and told them that nobody should deliver outside of the health center…the people were encouraged to build the maternity waiting home at the same time the awareness was going all over the entire communities.

Successful strategies included strong ties with the county level health team supporting the primary health clinic, community management teams, and strong support from Chiefs/Headmen and working with communities to develop strategies on how to adopt the intervention and its routine use for their particular context.

In many of the successful MWH communities, the TBAs were the key stakeholders in supporting use of the model, increasing community awareness, and working collaboratively with the health facility staff. Successful MWH models engaged all members of the community for the on-going sustainability of the model. One healthcare provider said: “The TBAs… went into all the communities and told pregnant women about the waiting home and asked them to come and wait before delivery … some staffer of the clinic also help to spread news of waiting through face to face discussions as well as community meetings.*”*

A community participant noted: “Awareness of the waiting home was also spread by the aid of community radios talk shows, health educators, and sometimes the certified midwife herself can go on the radio.”

The most successful MWHs included significant community engagement in the planning phases, widespread community awareness through community mobilization, and a plan for sustainable self-governance. Notably, some of the most successful MWHs in terms of utilization were not those with the most amenities–instead they were the homes in which the community was significantly invested.

#### Community ownership and governance

Successful, sustainable MWH models invested time and effort in eliciting community input, prioritizing community involvement, and taking steps to increase community awareness through a number of mechanisms. Sustainable models were self-funded by communities or communities were mobilized to contribute raw materials and/or land to the process. Similarly, implementing partners often worked with community groups to identify how to assimilate the MWH model to the primary health facility and services as noted by this participant: “The contractors were helped by the community through the molding of bricks, the provision of sand, gravels, cooking and at the same time drawing water.*”* Another participant said: “When we started building the MWH, one elder in the town gave a tree. When the people brought the project, we fixed bricks, we hauled sand, and gravel. Even the midwives and town citizens [contributed to the] work because we were in need.”

One example of self-governance was stated this way by a community member:

Traditional birth attendants come to [the MWH] to do cleaning up of the place. Secondly, [the traditional midwives decided] in a meeting to make a garden for the home since October 2017. Additionally to that…they set up a committee to help [supervise] the affairs of the home…where each community or town was responsible for cleaning up the home.

In communities where community engagement was done during the planning process, there was capacity building with communities on how to manage the MWH following project completion by the funder/implementer resulting in plans for sustainability. Community engagement was a critical component throughout the process from planning to management of the MWHs.

### Challenges

The data also revealed several challenges and barriers to sustainability and scale-up of MWHs in Liberia. The main themes challenging MWHs included: 1) food security; and 2) inadequate capacity.

#### Food security

The availability of food at the MWHs was one of the most common challenges given by communities. The vast majority of health facilities encourage women to bring food with them for their stay at the MWH, but women and family members found this to be challenging. Several reasons were given for this as one community member noted: “One of the things that scares away the big belly is the lack of food.” For a woman to bring food with her to a MWH, she must take away food that would otherwise be part of her family’s food supply, something few mothers in resource-constrained households are willing or able to do.” One healthcare provider described the impact of food insecurity on the use of the MWH in her community: “The major concern about this waiting home is the lack of food, which is causing most of the women to stay away.”

#### Inadequate capacity

Additionally, some of the most successful MWHs, in terms of attracting women to stay at them, now face the challenge of inadequate capacity, causing the MWH to become overcrowded with women. One healthcare provider noted: “This building is very small for us…sometimes we have two to three pregnant women in labor at the same time [and] no space.” As more and more women are receiving the message that a facility delivery is the safest and best for the mother and newborn, facilities were struggling with increased capacity at the MWHs. A TBA said: “The facility is very important to us in the area, but at first, we didn’t know the importance. That’s why it is small like this.…they can be sometimes six in numbers and room cannot host all of them at once, so it is causing us some embarrassment.”

## Discussion

Maternity waiting homes are not an isolated intervention. Rather, successful models become an integrated component of existing health system strengthening efforts. Our findings on sustainability mirror the key factors from a thematic synthesis of successful implementation of MWHs in LMICs including the importance of community engagement [23]. Barriers to sustainability such as food insecurity, lack of community awareness, and lack of development of a self-governance model corroborate results from several other studies on MWHs conducted in Sierra Leone, Nicaragua, and Ethiopia [24–26].

Only two articles in the literature provide examples of country wide scale-up [27,28]. In Peru, MWHs were scaled up to 390 facilities but little detail is given on influential factors other than incorporating MWHs as part of a ministry of health strategy [28]. The second example is from Cuba where 15 MWHs were introduced in 1962 to increase facility delivery. The number of homes there expanded to 327 by 2011 [27]. With its nearly 50-year history, these MWHs have been identified as a cost-effective approach to increasing facility delivery and decreasing maternal and newborn mortality in Cuba [27].

The value placed on MWHs to improve maternal health by participants in our study was high, adding to the successful adoption of the intervention and the wide-spread scale-up across the country. The positive response to MWHs generated enthusiasm about the intervention that positively influenced scale-up [29]. Nearly 30% of MWHs were implemented by local communities.

Because this was a country-wide study to examine the successes, challenges, and barriers to sustainability and scale-up of MWHs in Liberia, all MWHs within the country were included. The data collected from participants living in areas where MWHs were open and functional as well as communities where MWHs were under construction, re-purposed or abandoned allowed us to identify successful strategies as well as factors/challenges that contributed to failure to use the MWHs for their intended purpose adding depth to our findings.

By using multiple sources for data collection, we were able to develop a comprehensive understanding of the evolutionary development of MWHs, the role of MWHs to improve maternal health from a community and healthcare provider viewpoint, and document the successes, challenges, and barriers to sustainability and scale-up of MWHs in rural Liberia. This approach to data collection provided a deep understanding of the cultural and social context as well as visual and quantitative data.

Success factors for scale-up in Liberia included strong local and county leadership, active engagement of target communities at all timepoints, and self-governance. The political will and commitment of the government of Liberia to integrate MWHs into existing health system strengthening efforts as a way to reach women living farthest from a health facility additionally contributed to the successful scale-up in 14 of the 15 counties in the country. Public policy development by the Liberian Ministry of Health enhanced the scale-up of MWHs in Liberia [17]. In the MOH Annual Report (2013) the modest improvement in skilled attendance at birth was attributed to the scale-up of MWHs in the country [30]. In 2014, MWHs were listed as a strategy to strengthen and scale-up bi-directional referral systems in the Liberia Community Health Road Map [31]. These policies brought attention to funders and NGOs conducting development work within Liberia. Additionally, USAID, the funding agency for the original study, identified MWHs as one of several community based high-impact practices to improve maternal and newborn health in Liberia in their final project evaluation [32].

### Limitations and strengths

There are several limitations to this study. First, focus group participants were recruited through a convenience sample, potentially not being a representative sample of the community as a whole and resulting in bias. Nevertheless, it is our belief that the variety and breath of different locations across the country contributes to diverse representation. Second, focus groups included both women and men as well as chiefs and community leaders representing a power differential between members. This may have contributed to members not feeling comfortable to express their honest and personal opinions on the topic. While this potential limitation exists, the diversity of perspectives from a wide variety of individuals and the use of multiple methods for data collection strengthens our findings.

## Conclusion

Evidence from this study provides insights into one country’s strategy for scaling up a global health intervention to improve maternal outcomes. Thriving MWHs faced on-going challenges to sustainability such as food scarcity and inadequate capacity. Due to lack of resources or other constraints, local communities may become disheartened when efforts fail to complete the building of a MWH. Partnerships between funders and communities have the potential to increase scale-up with both partners contributing to the final outcome [23]. Findings can be applied at global and country levels to support policies and practices for the future scale-up of maternity waiting homes.

## Supporting information

S1 Appendix Interview guide for healthcare providers associated with maternity waiting homes. (DOCX)

S2 Appendix Focus group guide for community members. </SI_Caption
(DOCX)
