# Supplementary material for: Maternity waiting homes in Liberia: Results of a countrywide multi-sector scale-up
Source: PLoS One. 2020 Jun 23;15(6):e0234785. doi: 10.1371/journal.pone.0234785 (PMC7310707; doi:10.1371/journal.pone.0234785)
Supplement: S1 Appendix — (DOCX) [file pone.0234785.s001.docx]

**Interview Guide for Healthcare Providers Associated with Maternity Waiting Homes**

Facility Name: _____________________

Official Position: ___________________

Location: ___ National ___ County-level 🡪 County Name: __________

Location of Maternity Waiting Home (in relation to health clinic):__________________

Date: _______ Month _______Day _______ Year

Provide background on our project.

Thank you for taking the time to meet with us today. We are interested in learning more about maternity waiting homes in Liberia and how they are functioning. Please take your time and tell us as much as you can. Let me start with a few basic questions:

1. When did this maternity waiting home open? ________ (year)
2. How many beds does this MHW have? ________
3. Do you keep any records on the women who stay at the MWH?
4. Who usually accompanies the woman from her village?
5. Do you allow companions to stay with the women?
6. Do you allow children to stay with the women?
7. Can you tell me how many women stay here in an average month? ___________
8. Do you have any idea how long the average stay is? ______(days)
9. Do women pay to stay at the MHW? Yes / No / Don’t know
   1. If yes 🡪 How much? $_____
10. What supplies are provided?
11. Do any women stay here post-partum? Yes / No / Don’t know
12. Do you keep a record of how many women stay post-partum?
13. Do you think the MWH affects how the community sees the health facility? How, or why not? Can you give us some examples?
14. How do you think the MWH affects relationships between providers? (Probe: what about between traditional providers and western providers? What about between MWH providers and providers at the health center / hospital?)
15. How do you think the MWH affects delays in care-seeking?
16. What would you say are the main challenges to sustainability for this maternity waiting home?
17. Can you tell us about any successful models you know about for sustainability of maternity waiting homes?
18. What are the criteria that are required for women to be able to use the MWH? If yes, what are these?
19. Can you tell us what you think about demand for beds versus how many beds you have? In other words, are you able to meet the needs around here or do you feel like either you have too many beds or you are falling short? If the MWH is full and more women arrive, what do you do?
20. Can you tell us about what health education / topics are provided to women who stay at the MWHs? Are these provided on a regular basis? Do you keep a schedule of when these are offered?
21. Does the MWH have any income generating activities? If yes, tell us what you/the community are doing.
22. Can you tell us about how transfers and referrals are handled?
23. What do you think MWHs – or this one in particular - are doing really well?
24. Do you think MWHs – or this one in particular - could be improved?
